# The Influence of Extracorporeal Membrane Oxygenation on Antibiotic Pharmacokinetics

**DOI:** 10.3390/antibiotics12030500

**Published:** 2023-03-02

**Authors:** Gregory J. Peitz, Daryl J. Murry

**Affiliations:** 1 Nebraska Medicine, Nebraska Medical Center, Omaha, NE 68198, USA; 2 Department of Pharmacy Practice and Science, University of Nebraska Medical Center, Omaha, NE 68198, USA; 3 Clinical Pharmacology Laboratory, Department of Pharmacy Practice and Science, University of Nebraska Medical Center, Omaha, NE 68198, USA; 4 Fred and Pamela Buffett Cancer Center, University of Nebraska Medical Center, Omaha, NE 68198, USA

**Keywords:** extracorporeal, pharmacokinetics, pharmacodynamics, antibiotics

## Abstract

Extracorporeal membrane oxygenation (ECMO) is becoming increasingly utilized to support critically ill patients who experience life-threatening cardiac or pulmonary compromise. The provision of this intervention poses challenges related to its complications and the optimization of medication therapy. ECMO’s mechanical circulatory support is facilitated via various devices and equipment that have been shown to sequester lipophilic- and protein-bound medications, including anti-infectives. Since infectious outcomes are dependent on achieving specific anti-infectives’ pharmacodynamic targets, the understanding of these medications’ pharmacokinetic parameters in the setting of ECMO is important to clinicians. This narrative, non-systematic review evaluated the findings of the most recent and robust pharmacokinetic analyses for commonly utilized anti-infectives in the setting of ECMO. The data from available literature indicates that anti-infective pharmacokinetic parameters are similar to those observed in other non-ECMO critically ill populations, but considerable variability in the findings was observed between patients, thus prompting further evaluation of therapeutic drug monitoring in this complex population.

## 1. ECMO Background

Extracorporeal membrane oxygenation (ECMO) is a mechanical circulatory support (MCS) modality that is used for acute cardiopulmonary collapse [1]. The complete ECMO circuit is a type of cardiopulmonary bypass that is able to facilitate oxygen delivery to tissues in the setting of significant cardiac or respiratory compromise while also removing carbon dioxide produced during cellular respiration [2]. Initially an intervention most often utilized in non-adult populations, this advanced emergent life-saving support has increasingly been employed following the benefits demonstrated in the Conventional Ventilator Support versus Extracorporeal Membrane Oxygenation for Severe Acute Respiratory Failure (CESAR) study [3], and has seen continued supporting evidence in acute respiratory disorders [4], as a bridge to heart transplant or a durable MCS device [5,6], or following cardiac arrest and cardiogenic shock [7,8]. It has also shown promise as a method to improve hypoxic-related outcomes during liver transplantation [9]. 

## 2. Trends in ECMO Utilization

ECMO utilization has increased exponentially in health care institutions across the world, with over 100,000 adult cases officially registered in the Extracorporeal Life Support Organization (ELSO) database as of October 2022 [10], making the impact of pharmacologic administration with this complex support paramount to understand. While it offers new management options for severely ill patients, ECMO facilitation poses a multitude of possible complications. As ECMO is used with an increasing frequency, further efforts to understand its challenges will help practitioners provide more specific care. The intention of this narrative review is to discuss ECMO support’s potential impact on commonly used antimicrobials and to examine available literature to optimize therapeutic regimens in the context of ECMO use. 

## 3. Infective Complications during ECMO Support

### 3.1. Infectious Sources/Locations

While ECMO is increasingly utilized in emergent, life-threatening scenarios, the intervention poses numerous risks to its recipients, including concerns related to hemorrhage, thrombosis, neurologic injury, and infectious complications which are the second most frequent setback after hemorrhagic issues [11,12]. In addition to focusing on infectious complications after ECMO cannulation, it is equally important to recognize that infections may be present prior to the placement of an ECMO circuit. Patients who are cannulated for venovenous (VV) ECMO may be receiving cardiopulmonary support for acute respiratory distress syndrome (ARDS) secondary to bacterial or viral pneumonia [13,14]. Patients supported by venoarterial (VA) ECMO may likewise be impacted by infectious insults such as sepsis-associated cardiomyopathy or viral myocarditis [12,13]. Positive microbiological cultures have been reported prior to ECMO cannulation in 31.6% of VV ECMO recipients, 8.8% in VA ECMO, and 7% in extracorporeal cardiopulmonary resuscitation (ECPR) [12].

Nosocomial infections are also common in the setting of ECMO, with notable risk factors including the concomitant use of invasive mechanical ventilation, older age, prolonged hospitalization, underlying comorbidities, concomitant utilization of mechanical devices, and an extended duration of ECMO support [14]. Data indicates that up to 65% of adults receiving ECMO may develop an infection while on therapy, with VV ECMO recipients being the most commonly affected [14,15]. The expected incidence of infection while on ECMO ranges from 12–75 events per 1000 ECMO cannulation days [14,15,16]. The most common sources of nosocomial infection that develop while on ECMO support are ventilator-associated pneumonia (VAP), blood stream infections (BSIs), and catheter-associated urinary tract infections (UTIs), with incident rates of 24, 20, and 12 events per 1000 ECMO days, respectively [14]. Mediastinitis can be an issue in patients receiving VA-ECMO and cannula-related infections have also been noted in this population [15,17,18]. Lastly, the ECMO circuit itself, including the cannulas or oxygenator, can become colonized with microorganisms from alternative infectious sources leading to a difficult-to-treat infection [17,18,19].

### 3.2. Common Pathogens

Nosocomial infections during ECMO support are secondary to similar microorganisms responsible for infectious insults in other critically ill patients. Bloodstream infections are often caused by *Staphylococcus aureus*, *Enterococcus* species, and fungal pathogens, whereas respiratory infections are due to *Klebsiella* species, the Enterobacteriaceae family, and *Pseudomonas aeruginosa* [12]. Patients who present with bacteremia prior to ECMO cannulation have twice the odds of being infected with a multidrug-resistant microorganism after they are initiated on ECMO support [20]. Fungal infections do occur during ECMO support, but their incidence is no greater than observed in other critically ill patients and their development is often delayed following cannulation [21,22]. 

### 3.3. Observed Outcomes

The risk of death may increase by up to 63% following a nosocomial infection while on ECMO [14]. Independent risk factors for mortality following an infectious insult include increasing age, presence of membrane oxygenator microorganism colonization, and concurrent receipt of renal replacement therapy (RRT). An infection exacerbated by the development of sepsis portends worse outcomes in this patient population. Sepsis has been demonstrated as an independent predictor of failure to discharge despite being successfully decannulated from ECMO [23]. Regardless of the presence of ECMO, sepsis is a major contributor to overall mortality for patients admitted to an ICU, with mortality ranging from 20–50% [24,25]. The benefits of early and appropriate antibiotic initiation in patients with sepsis are well known and unequivocal [26]. Substantial increases in mortality and morbidity have been demonstrated for every hour of delay in initiation of appropriate antibiotic therapy [27].

## 4. ECMO Configurations 

Critically ill patients present with a host of compelling factors that can impact the pharmacodynamics of medications including an inflammatory response, capillary leak, and resultant edema. These physiologic changes can be further compounded during the facilitation of ECMO support [28]. In addition to these alterations in inflammatory mediated fluid shifts, patients on ECMO are also prone to alterations in their blood pH, which can impact a medication’s ionization and ultimate distribution into tissues. Furthermore, the upregulation of the renin-angiotensin system in the setting of ECMO-induced nonpulsatile flow can change the ratio of fluids in the body fluid compartments [28].

The insertion of ECMO into a critically ill patient also creates challenges to ensuring appropriate medication delivery. The ECMO circuit consists of several components, including the circuit tubing, blood pump, membrane oxygenator, and heat exchanger [29]. An ECMO circuit can be configured to support a critically ill patient in multiple anatomical arrangements. VV ECMO consists of a drainage cannula from a venous access site with return blood being delivered through a venous access point. VA ECMO also incorporates de-oxygenated blood from a venous cannula but returns the oxygenated blood from the device through an arterial access site. Both VV and VA can be arranged either peripherally or centrally depending on a patient’s clinical situation. In any configuration, the de-oxygenated blood is circulated within large bore cannulas via a mechanical pump through a circuit that includes an in-line membrane oxygenator and gas blender combined with a heat exchanger and a filter before the re-oxygenated blood is introduced back into a patient’s systemic circulation [2]. The oxygenator is partnered with a blender to both provide oxygen directly to circulating blood as it crosses the apparatus, while it also simultaneously removes carbon dioxide from the venous sample. Oxygenators can also regulate body temperature as they are often incorporated with a heat exchanger. As blood moves through the configuration, pre-oxygenator and post-oxygenator pressures are continuously monitored to assess any disturbances in blood flow throughout the system and arterial blood gases can be drawn directly from the circuit to objectively identify oxygen delivered via the device. Two types of pumps are available to facilitate blood flow: roller pumps or the newer, more frequently used centrifugal pumps. The circuit’s tubing and oxygenator include hollow fiber tubes which are composed of polyurethane, polyvinyl chloride, silicone rubber, or polymethylpentene [2]. The cannulas may be coated with heparin or non-heparin polymers which are utilized to minimize platelet activation or inflammatory mediation induced during the cannulation procedure [2]. 

## 5. Challenges with Medication Therapy

The provision of ECMO support has created new dosing challenges as the device has been shown to increase volumes of distribution (Vd) and may alter drug concentrations. Specifically, ECMO circuits have demonstrated that lipophilic medications that are extensively protein bound may be sequestered in the ECMO device [30,31]. The circuit tubing and oxygenator materials can impact drug sequestration as lipophilic medications can become adsorbed which leads to increases in Vd. This increase in Vd and the lipophilic conduit may alter the pharmacokinetics (PK) and pharmacodynamics (PD) of commonly used intensive care unit (ICU) medications [32,33]. Studies have demonstrated the impact of ECMO on drug binding and altered dose requirements in this exposed population. For instance, fentanyl, propofol, and midazolam, which are all highly lipophilic medications, have all been shown to adhere to the device tubing in ex-vivo models [30,34]. 

Antimicrobial PK and PD are particularly important when choosing appropriate antibiotic doses in the critically ill population. However, most antibiotic dosing recommendations are obtained from healthy subjects, creating limited data in patients with altered physiologies, and abnormal volumes of distribution. Ex-vivo-based research in ECMO circuits has demonstrated that antibiotics with a high degree of lipophilicity and protein binding may be sequestered in the ECMO device [30,33]. The binding of these antibiotics could then impact the overall drug delivery to the patient and intended microbial target, and subsequently lead to worse outcomes. As the surviving sepsis guidelines strongly advocate for empiric broad-spectrum antibiotics in sepsis, it is also recommended to optimize antimicrobial dosing to improve outcomes of patients with severe infection [35]. This may require higher than normal loading doses of antibiotics to avoid underdosing early in sepsis due to altered volumes of distribution given the physiology of disease and ensuing fluid resuscitation [36].

Due to an increasing rise of ECMO utilization, then, there is a critical need to understand how this intervention alters patient specific PK parameters and PD response to optimize antibiotic dosing. 

## 6. Antimicrobial PK Literature Review 

While it is understood that appropriate antibiotic therapy is necessary for successful intervention, ensuring this outcome is not always fully realized. Although the interactions between administered antimicrobials and the ECMO circuit have been evaluated in ex-vivo models, robust prospective in-vivo PK data to guide effective antimicrobial dosing is still limited. Review of available literature on all anti-infective medications could be comprehensive, but rather than focus on all available data from small single patient reports or retrospective reviews, the intent of this non-systematic review is to provide a summary of data from those anti-infectives most impactful to the care of critically ill patients with multi-drug resistant organism (MDRO) risks. The optimal dosing of beta-lactam antibiotics in ECMO support is of particular interest since these antimicrobials often serve as the primary empirical or definitive treatment for serious infections in the critically ill population. 

## 7. Ex-Vivo Studies

The concern regarding the efficacy of antibiotics in the setting of ECMO first originated with ex-vivo circuits demonstrating the impact that an ECMO circuit can have on various classes of medications, including antibiotics. These ex-vivo models aimed to identify sequestration of various medications in an ECMO circuit over a 24 h period as compared to a control circuit [34,37]. Initial results postulated that drugs with low Vd and high protein binding may indeed be impacted by the ECMO circuit [30,31,38]. Although ex-vivo studies may be useful in understanding how drugs interact with an ECMO circuit in isolation, the results of this pre-clinical data should be cautiously translated into critically ill patients, as the reported extraction is only related to the absorption of the ECMO component materials and does not account for tissue uptake, in-vivo metabolism, or other physiologic changes that can occur in the setting of critical illness. 

## 8. Prospective In-Vivo Data

Unlike ex-vivo modeling, prospective in-vivo studies provide patient-centered data to identify medications’ pharmacokinetic profiles and, thus, a more specific understanding of pharmacodynamic attainment. When interpreting any anti-infective’s PK and PD profile, it is imperative to first understand an agent’s specific definition of the optimal pharmacodynamic target. Likewise, understanding how a medication’s serum concentration relates to toxicity is also warranted. A summary of these targets and thresholds can be seen in Table 1. The anti-infectives discussed below are chosen based on the pragmatic application within the clinical setting, specifically focusing on those therapies often initiated in the setting of presumptive or definitive infection during a critical illness. 

### 8.1. Cefepime

Cefepime has a logP (octanol-water partition coefficient) of −0.1 with approximately 20% protein binding [39]. It has been characterized in various ECMO settings. Kois et al. [40] recently evaluated six critically ill patients receiving ECMO support, all of whom were receiving high doses of cefepime therapy (2 g every 8 h extended over 3-h). A two-compartment model fitted the data best with median parameter estimates as follows: clearance (CL) 5.99 L/h, and Vd 10.08 L. The investigator’s simulation for various cefepime dosing regimens concluded that a 2 g every 8-h regimen successfully reached the desired target of ≥70% ƒT > MIC at 4 µg/mL and 8 µg/mL with either a 30 min or 3-h infusion. A 3 h infusion was necessary to reach the specified threshold of a minimum inhibitory concentration (MIC) of 16 μg/mL. Simulations demonstrated that a 2 g every 12-h regimen would obtain a ≥70% ƒT > MIC for MICs of 4 µg/mL or 8 µg/mL in five out of six patients, but a 1g every 12-h regimen would not be able to successfully reach the prespecified target. Simulations of the high dose regimen of 2 g every 8 h were described as more likely to result in trough concentrations exceeding a neurotoxic threshold [41,42,43], however.

In the more recent ASAP ECMO trial [44], which is the largest, most robust effort to date on prospective PK characterization in commonly prescribed critical medications, cefepime PK parameters were reported based on six individuals receiving a median total daily dose of 3.3 g. The median PK findings are as follows: CL 2.42 L/h, Vd 17.91 L, C_max_ 85.03 mg/L, C_min_ 32.25 mg/L, T_1/2_ 6.2 h, and AUC_0–24_ of 1040 mg*h/L. These results demonstrate an apparent higher Vd and T_1/2_, but lower CL and C_min_, in contrast to other critically ill patients not receiving ECMO as examined by Kassel et al. [45]: CL 10.6 L/h, Vd 32 L, and C_max_ 81.5 mg/L, C_min_ 5 mg/L, T_1/2_ 2.4 h. Despite obtaining median values above the desired target threshold of 8 mg/L in the ASAP ECMO study, interpatient variability with that dosing regimen was noted, as the interquartile range for the trough value was 4.92–48.31 mg/L. Results from Kois et al. may strengthen the argument for utilizing a 2 g every 8-h approach, particularly in the setting of MDRO, but this regimen may increase unintended side effects. 

### 8.2. Meropenem

Meropenem is a time-dependent carbapenem that is routinely utilized to empirically or definitively treat multi-drug resistant organisms. With a logP of −0.6 and protein binding of approximately 2% [46], meropenem’s profile indicates it would likely not be sequestered by the ECMO components. Of evaluated antibiotics, though, it has extensively been evaluated in the ECMO setting. A 2015 case–control study by Donadello et al. compared 27 ECMO recipients to matched controls who were receiving meropenem [47]. Using a one-compartment-based PK model, the investigators did not find any differences in meropenem’s V_d_, T_1/2_, or CL compared to a placebo. Of note, however, the study highlighted a considerable inability to achieve target meropenem concentrations in both the ECMO and control cohorts. The most complete PK data in ECMO therapy was reported in the recent ASAP ECMO study in 18 patients during which meropenem had median C_max_ of 59.36 mg/L, C_min_ 6.25 mg/L, AUC_0–24_ of 495 mg*h/L, T_1/2_ 2.77 h, Vd of 29.8 L, and a CL of 7.2 L/h. Those receiving RRT in the study had an increase in C_max_, C_min_, AUC_0–24_, T_1/2_, and Vd, but a reduction in overall CL compared to those not receiving RRT. The C_max_ observed in this study is considerably higher than parameters reported by the EXPAT study [48]: (C_max_ 7.7 mg/dL, C_min_ 4.6 mg/dL), despite similar meropenem doses in each trial (3.4 g every 24 h in ASAP ECMO vs. 3 g every 24 h in EXPAT). Similar trough concentrations were seen in the ASAP ECMO study compared to the DALI study [49] (6.25 mg/L vs. 5 mg/L). Although the median trough values while on ECMO potentially support using standard meropenem dosing in this population, it should be noted that there was considerable variation in those observed values. The interquartile range for the C_min_ in the aggregate population ranged from 0.2–19.9 mg/L with a between-subject coefficient of variation (CV) ranging between 29.32–66.97% depending on the modality of ECMO (VV or VA) and presence of RRT. 

### 8.3. Piperacillin

Piperacillin is often used in combination with tazobactam as an empiric anti-pseudomonal agent in the intensive care setting. It has a logP of 0.5 and is a moderately protein-bound drug (20–40%) [50,51]. Piperacillin has had numerous analyses of its pharmacokinetic parameters in the setting of ECMO including a case–control study evaluating 14 subjects receiving piperacillin/tazobactam by Donadello et al. [47] Similar to the study’s meropenem data, there was no difference observed in piperacillin/tazobactam in the ECMO or control groups’ PK parameters. To date, ASAP ECMO investigators provide the most robust PK data. In the 27 individuals evaluated in that analysis, piperacillin had a median C_max_ of 132.6 mg/L, C_min_ 13 mg/L, AUC_0–24_ of 937.5 mg*h/L, with a T_1/2_ of 2.06 h, Vd of 28.1 L, and CL of 10.9 L/h in the aggregate population. Other PK evaluations in ECMO populations include an assessment of 48 patients by Kuhn et al. [52], of which 14 patients were on ECMO and received 4.5 g of piperacillin/tazobactam every 8 h during therapy. In that PK analysis, the individuals on ECMO had significantly lower piperacillin concentrations than the 34 patients not on ECMO (32.3 vs. 52.9 mg/L, *p* = 0.029). Of note, the interquartile range of the trough in the ASAP ECMO trial illustrated a much larger range of values than seen by Kuhn et al. Both assessments of C_min_ values in the ASAP ECMO study as well as data from Kuhn et al., demonstrate higher trough concentrations than the assessment in the DALI study. The ASAP ECMO evaluation yielded similar AUC_0–24_ when compared to the DALI study (937.5 vs. 1124.2 mg*h/L). 

### 8.4. Vancomycin

Vancomycin is the most widely used antimicrobial agent for methicillin-resistant staphylococcus aureus (MRSA). It is one of few antibiotics used that has a readily available laboratory tool to assess therapeutic achievement and is often guided by therapeutic drug monitoring. It has a logP of −3.1 and is 50% protein-bound [53]. Retrospective studies have evaluated traditional vancomycin dosing regimens in the setting of ECMO, and results have been inconsistent, with findings raising concerns for both subtherapeutic and supratherapeutic therapy in the population [54,55]. It has been studied in several prospective ECMO studies as well, with the ASAP ECMO supplying the most complete data.

Results from the ASAP ECMO study included 22 patients who received a median of 1.8 g of vancomycin every 24 h. In the analysis, the median AUC_0–24_ observed was 386.5 mg*h/L in the total ECMO cohort, with lower values seen in those receiving RRT (AUC_0–24_ 367 mg*h/L) than those not on RRT (AUC_0–24_ 505 mg*h/L). These values were lower than the median value observed in the DALI study (AUC_0–24_ 655 mg*h/L)_._ Other PK parameters of note for those in the ASAP ECMO trial include a C_max_ of 41.08 mg/L, C_min_ 11.6 mg/L, Vd 31.9 L, and CL of 3.79 L/h. With the central tendency in this population lower than the desired threshold of an AUC_0–24_ ≥ 400 mg*h/L, and the wide variability seen, practitioners should consider administering loading doses with therapeutic monitoring engagement to ensure pharmacodynamic success when using vancomycin. 

### 8.5. Linezolid

Linezolid has a logP of 0.9 with 31% protein binding [56]. Standard doses of linezolid (600 mg every 12 h) have been evaluated in an ECMO setting by de Rosa et al. [57] (3 patients) as well as within the ASAP ECMO study (1 patient) to evaluate pharmacokinetic parameters. The analysis from de Rosa et al. yielded similar PK parameters as observed in other non-ECMO critically ill patients as examined by Simon et al. [58] with a C_max_ of 16.6 mg/L vs. 21.9 mg/L, AUC_0–24_ 156.6 mg*h/L vs. 63.4 mg*h/L, Vd of 38 L vs. 37.7, and CL of 8.7 L/h vs. 7.2 L/h. The one patient within the ASAP ECMO study concluded similar PK findings. Because linezolid has time-dependent antibiotic activity with a modest concentration-dependent kill characteristic for a target AUC_0–24_ goal of 80–120, these findings pertaining to standard dosing in an ECMO setting support standard dosing of linezolid. In contrast to standard dosing, PK data is available as well for higher dose regimens while on ECMO therapy. In a study of 19 individuals receiving linezolid 600 mg every 8 h while on ECMO, Kuhn et al. [52] reported a median trough value of 8.6 mg/dL suggesting a higher likelihood of toxicity, as linezolid-induced thrombocytopenia has been reported with C_min_ values greater than 7 mg/L [59]. Therefore, standard dosing regimens should continue to be employed in the setting of ECMO until additional studies can be performed to assess the risk of toxicity of non-conventional linezolid dosing strategies. 

### 8.6. Fluconazole

Fluconazole is a neutrally charged azole with a logP of 0.4 and 11% protein binding [60]. It can be commonly used for *C. albicans* fungemia in the critically ill. Prior to the ASAP ECMO study, evaluation of its PK in human subjects during ECMO was extremely limited. Shekar and colleagues, evaluated 10 patients with a median dose of approximately 7 mg/kg daily. The aggregate fluconazole PK findings were as follows: C_max_ 14.64 mg/dL, C_min_ 6.79 mg/dL, AUC_0–24_ 232 mg*h/L, T_1/2_ 15.2 h, Vd 59.86 L, and CL 3.08 L/h. The PK parameters were heavily influenced by the presence of RRT in this population, as those without RRT had a much higher C_max_ (30.46 vs. 12.39 mg/L), C_min_ (20.14 vs. 6.03 mg/dL), and AUC_0–24_ (592 vs. 206 mg*h/L) compared to individuals who received RRT. This was likely influenced by the differences seen in clearance between the two groups, as those who received RRT had higher Cl (3.46 vs. 1.08 L/h) and shorter T_1/2_ (11.3 vs. 24.4 h) when compared to those without RRT. The ASAP ECMO study results demonstrated similar Vd values, C_min_, and AUC_0–24_ when compared to data compiled by Boonstra et al. [61] in 49 non-ECMO critically ill patients. It should be noted, however, that the non-ECMO patients received lower doses per day (1.4–3.8 mg/kg/day) than those individuals in the ASAP ECMO study, thus making it harder to translate standard dosing effectiveness across populations. 

### 8.7. Voriconazole

Voriconazole is an alternative azole that has a broader spectrum of activity to fluconazole and may be used empirically in infectious situations concerning resistant fungi [62]. It has a logP of 1 and has 58% protein binding [63]. Voriconazole has been most extensively studied in an ECMO setting by Van Daele et al. [64], who retrospectively reviewed voriconazole concentrations in 69 adult patients. In their review, they found no significant differences between voriconazole C_min_ values when drawn during ECMO therapy versus those drawn off ECMO therapy (2.4 mg/L vs. 2.5 mg/L, *p*-value of 0.58). Of note, there was a high inter-subject variability seen with 59% CV and 49% CV in the ECMO and non-ECMO populations, respectively. Other evaluations of voriconazole PK on ECMO therapy have come from single patient studies. The ASAP ECMO group evaluated 1 patient prospectively and other reports are based on single observances as well [65,66,67,68]. These analyses have demonstrated varying PK results with C_max_ ranges from 3.85–16.7 mg/L and C_min_ values of 0.8–13.28 mg/L, which may be reflective of the doses used in each study, severity of illness, or other confounding factors such as drug interactions or end-organ dysfunction. Prospective PK studies have recently been undertaken in non-ECMO patients; however, these trials have been conducted in patients with varying degrees of Child–Pugh liver dysfunction [69,70]. With the limited and variable prospective PK findings in ECMO, there may not be sufficient evidence to conclude on optimal voriconazole dosing in this population at this time. 

### 8.8. Echinocandins

The echinocandins are often used in the critical care setting for empiric or definitive treatment of non-C. albicans infectious insults [62]. All three of the available echinocandins: caspofungin, micafungin, and anidulafungin, have been evaluated in the setting of ECMO [44,60,67,71,72], but caspofungin has the most complete PK data in human subjects. Consistent with the other echinocandins, caspofungin is highly protein-bound (95%) [73], but has a low logP (−3.8) [74]. In a study by Wang et al. [75], caspofungin was evaluated in 12 patients on ECMO and 7 patients without ECMO following lung transplant. Following the administration of caspofungin 50 mg every 24 h in each group, investigators determined no significant differences in PK parameters between those patients who received ECMO versus those without ECMO: Cmax 16.7 vs. 17.9 mg/L, C_min_ 3.5 vs. 3.5 mg/L, Vd 3.22 vs. 3 L, CL 0.27 vs. 0.31 L/h, AUC_24–48_ 163 vs. 156 mg*h/L, or T_1/2_ 16.9 vs. 14.2 h. Despite receiving higher caspofungin doses in the ASAP ECMO study (80 mg every 24 h), the PK parameters observed were considerably different from those reported by Wang and colleagues. The ASAP ECMO study reported a lower C_max_ (7.86 mg/L), C_min_ (2.63 mg/L), and T_1/2_ (8.46 h), as well as AUC (112 mg*h/L), which was captured from 0–24 h instead of 24–48 h, as performed by Wang et al. When comparing this PK data during ECMO therapy to other studies of critically ill patients without ECMO, the available PK data is conflicting. Adembri et al. [76], evaluated 20 critically ill patients who received a traditional 70 mg loading dose followed by 50 mg daily of caspofungin. Characterization of PK values at day 4 revealed the following mean values: C_max_ 13.5 mg/L, C_min_ 3.24 mg/L, AUC 132 mg*h/L, and Vd 6.1 L. These findings were similar to those seen in the ECMO patients reported by Wang et al., but considerably different to the ASAP ECMO findings. In contrast to the findings of Adembri et al., van der Est and colleagues [77] presented PK data in non-ECMO critically ill patients, demonstrating values similar to the ASAP ECMO results. In the context of these mixed results, then, it is undetermined if ECMO contributes to any significant PK changes. 

## 9. Monitoring

The available data in prospective ECMO cases is consistent with PK findings observed in other critically ill patient populations, but the question about using standard dosing in this population remains unanswered. Results from the largest, most well-designed study to date in the ECMO population contains information from a limited number of patients, with the presence of confounders such as RRT. This heterogeneity amongst included patients is readily recognizable as there was considerable interpatient variability as demonstrated by the coefficient of variation noted for each anti-infective. The interpatient variability in these limited findings should raise concern, then, as many standard dosed anti-infectives will fail to reach optimal pharmacodynamic thresholds for clinical success, particularly in cases with more resistant organisms. This leaves an opportunity for discussion regarding the utility of the implementation of prospective PK monitoring. Certainly, this caveat would extend beyond those patients who receive ECMO, as patients in the critical care arena may have their medication PK parameters affected by other confounders. Results from the DALI [49] study illustrated the considerable inconsistencies in PD achievement seen with standard anti-infective dosing in the critically ill population and investigators in the BLISS trial [78] subsequently highlighted the opportunities for optimal patient care when PD targets are attained. Due to increasing frequency of ECMO utilization, and observed variations seen in the few prospective pharmacokinetic studies, implementation of prospective TDM should be considered throughout the course of antibiotic therapy in this complex setting. 

## 10. Ongoing Challenges in the ECMO Setting

There is little data available in the literature that addresses resistant organisms within the ECMO population, but challenges exist for all critically ill patients, with those individuals on ECMO at an equal or greater risk for failure. Future studies should evaluate antimicrobial PK and PD in this population to provide clinicians with objective data to improve patient outcomes

There are numerous challenges faced by future ECMO studies when evaluating PK parameters. First, the issues introduced by the ECMO circuit are certainly compounded by concomitant critical illness. PK and PD are already well known to be altered in this population, due to alterations in a patient’s serum protein levels, acid–base imbalances, and intrinsic altered volumes of distribution caused by pathophysiologic disturbances during critical illness [36,79].Furthermore, critically ill patients are more likely to have renal and hepatic abnormalities, reducing the clearance of some medications. Indeed, these confounders have been illustrated to impact the consistent obtainment of therapeutic drug concentrations in the intensive care patients, adding to the ambiguity of standard medication dosing in the population [49]. Certainly, patients on ECMO support are at an elevated risk of worsening organ function, but the impact on medication clearance has not been prospectively validated in adult patient models. As previously mentioned, current recommendations for most drug dosing guidelines are obtained from healthy subjects, creating limited data in patients with an altered physiology, or abnormal volumes of distribution. Presumptively, the binding of certain medications to the circuit and alterations in the pharmacokinetic profile of select drugs could subsequently lead to worse outcomes. 

Secondly, in addition to the difficulty with adjusting for issues related to critical illness, future studies are also impacted by the variation in delivery of ECMO support itself, particularly across various institutions with differing experience levels. Most studies including patients receiving ECMO are typically undertaken from single centers with small sample sizes that do not account for heterogeneity, mechanical ventilation delivery, and types of ECMO equipment used. Moreover, the expertise of practitioners may vary from center to center and even within a given institution, as well. Often studies involving ECMO patients may include both VV and VA patients simultaneously. While this may increase the sample size, it may worsen generalizability of the findings as the prognostication and challenges faced by each type of cannulation may be different. Even the decision on the timing to ECMO support initiation for a particular patient may vary greatly depending on an institution’s own guidance or what predictive model they may employ to aid with decision-making. Recent findings have perhaps made this evaluation of ECMO initiation more complex, as data indicates no improvement in outcomes with immediate ECMO deployment when compared to a delayed approach in patients with cardiogenic shock [80]. 

Lastly, to obtain the most objective unbiased data in this population, more prospective studies would be ideal. This presents a pragmatic challenge, due to the nature of the device employment. ECMO cannulation most often occurs during a period of significant patient compromise with little time to interact with a prospective patient. Additionally, due to their current medical condition, patients are frequently not able to cognitively or ethically comprehend or give consent to experimental techniques that are being introduced, and family members or legally authorized representatives may also be absent at the outset of ECMO cannulation. Due to the years that it may take to successfully enroll an adequate number of subjects to objectively answer a specific question in this population, ECMO techniques or equipment may have already changed, making the results from prospective studies less applicable in future care. Subsequently, future investigations should consider utilizing both pragmatic research designs and inclusion of large database queries to advance the knowledge in this field. 

## 11. Conclusions

In summary, ECMO is a life supporting modality that can be acutely employed in the setting of cardiopulmonary collapse. Its facilitation in the critically ill population introduces untoward complications and challenges, including the optimal provision of medication therapy, particularly during the application of anti-infective drugs. Based on the available prospective pharmacokinetic literature, standard dosing of anti-infective agents in the ECMO population appears to provide similar PK parameters compared to other critically ill patients not receiving ECMO. However, the observed variation in many of the medications’ concentrations poses questions regarding the need for prospective therapeutic drug monitoring to ensure optimal pharmacodynamic targets are achieved for all patients who receive ECMO support. 

## Figures and Tables

**Table 1:** Summary of anti-infective characteristics with target pharmacodynamic and toxicity parameters.

Anti-Infective Agent	LogP	Protein Binding	Target Efficacy Parameter	Toxicity Threshold Parameter
Cefepime	−0.1	20%	45–100% *f*T ≥ MIC (C_min_ ≥ 8 mg/L)	C_min_ > 20 mg/L
Meropenem	−0.6	2%	50–100% *f*T ≥ MIC (C_min_ ≥ 2 mg/L)	C_min_ > 45.5 mg/L
Piperacillin	0.5	20–40%	50–100% *f*T ≥ MIC (C_min_ > 16 mg/L)	C_min_ > 361 mg/L
Vancomycin	−3.1	50%	Total AUC_0–24_/MIC ≥ 400	Total AUC_0–24_ ≥ 700 mg*h/L
Linezolid	0.9	31%	Total AUC_0–24_/MIC: 80–120; ≥85% T ≥ MIC (C_min_ > 2 mg/L)	Total AUC_0–24_ > 300 Total Cmin > 7 mg/L
Fluconazole	0.4	11%	Total AUC_0–24_/MIC ≥ 55–100	Uncertain
Voriconazole	1	58%	Total C_min_ ≥ 1–2 mg/L	Total C_min_ ≥ 4.5–6 mg/L
Caspofungin	−3.5	95%	Total AUC_0–24_/MIC > 3000	Uncertain

Adapted from Abdul-Aziz MH, et al. Intensive Care Med. 2020; 46(6): 1127–1153.

## Data Availability

Data sharing not applicable.
